# Fractional Exhalation Nitric Oxide (FeNO) changes in cystic fibrosis patients induced by compound honey syrup: a pretest–posttest clinical trial

**DOI:** 10.1186/s12890-023-02787-9

**Published:** 2023-12-05

**Authors:** Saeed Sadr, Hanieh Tahermohammadi, Shahpar Kaveh, Ghamartaj Khanbabaee, Seyed Ahmad Tabatabaei, Rasool Choopani, Arian Karimi Rouzbahani, Nafise Fadavi, Shima Derikvandi

**Affiliations:** 1 Department of Pediatric Pulmonology, Mofid Children’s Hospital, Shahid Beheshti University of Medical Sciences, Tehran, Iran; 2 Chronic Respiratory Diseases Research Center, National Research Institute of Tuberculosis and Lung Diseases (NRITLD), Shahid Beheshti University of Medical Sciences, Tehran, Iran; 3 Persian Medicine Network (PMN), Universal Scientific Education and Research Network (USERN), Tehran, Iran; 4 Department of Traditional Medicine, School of Traditional Medicine, Shahid Beheshti University of Medical Sciences, Tehran, Iran; 5 Student Research Committee, Lorestan University of Medical Sciences, Khorramabad, Iran; 6 Rajaei Cardiovascular Medical and Research Center, Iran University of Medical Sciences, Tehran, Iran; 7 Faculty of Veterinary Medicine, Student University of Tehran, Tehran, Iran

**Keywords:** Cystic fibrosis, Compound honey syrup, FeNO, Persian Medicine

## Abstract

**Objective:**

To evaluate the effect of Persian medicine Syrup ‘compound honey syrup (CHS)’ on fractional exhalation nitric oxide (FENO) changes in patients with cystic fibrosis (CF).

**Study design:**

We conducted a before-after clinical trial on 70 CF patients. All patients received classical treatments for CF along with CHS (including honey, Ginger, cinnamon, saffron, cardamom and galangal), 5–10 cc (depending on the age and weight of patients) in 100 cc of warm boiled water twice a day, 30 min after meals. In this clinical trial, before and 12 weeks after the start of the CHS, FeNO test was evaluated.

**Results:**

From 70 patients were enrolled, 44 patients completed this 12-week course of treatment. At the end of the study, changes in FeNO was significantly different before and after treatment (*P*-value < 0.05). At the end of the study, no dangerous side effects of CHS was reported.

**Conclusions:**

This study revealed that CHS can be effective as a complementary and safe drug in the medication of CF patients.

## Introduction

CF is a chronic and progressive genetic disease that, as a multisystem disease, affects not only the lungs, but also the pancreas, liver, and digestive system [1]. CF is the most common fatal genetic disorder in white people. This disease occurs in 1 in 2000 to 1 in 3500 births [2]. With the advent of new treatments, the average expected survival in patients has increased dramatically from about 8 years for patients born in the 1960s to about 46 years for patients born in 2017 [3]. Cystic fibrosis is an autosomal recessive disorder arising from mutations in the cystic fibrosis transmembrane conductance regulator (CFTR) gene [4]. CFTR is a cAMP-regulated chloride (Cl −) channel chiefly located on the apical sides of epithelial cells inner coating exocrine glands, gut, and airway, where it initiating transepithelial salt and water transfer [5]. Mutated CFTR fails to supply the ionic drive necessary to be properly hydrated of the mucous sheet, leading to thick mucosa with concentrated mucins which collapses onto the submucosa as a first step upon chronic infection and organ disease [6].

Moreover, the thick and adhesive mucosa which is typical feature of the airways with inflammation in cystic fibrosis might block the diffusion of Nitric Oxide (NO) from the synthetic area in the mucous layer to luminal air [7]. NO is a free radical gas and a message carrier molecule which is released in the airway and is definable in blew out air. The physiological engagement of breathed out NO possibly includes regulation of antimicrobial activity, mediation of inflammation, ciliary activity, dilation of vessels and bronchus [8, 9].

Fractional exhalation of Nitric Oxide (FeNO) in CF patients is plummeted with normal or lung function reduction in comparison with non-CF control subjects, and lately it has been indicated that FeNO is inverse correlation with the lung-clearance index. The reason that FeNO is lowered in CF is still unclear, although hypotheses consist of locally trapped NO in the dense mucous lining characteristic of the CF respiratory tract, reduced production due to lack of NO synthase, rising NO consumption, or inadequacy in ciliary activity [10]. All of these hypotheses have direct or indirect correlation with roles of CFTR [11].

To ease the elimination of Thick lung secretions, medicines that reduce mucous density and plasticity are frequently applied in various lung disorders, and particularly in CF [12].

In addition to taking antibiotics in certain cases, the treatment for cystic fibrosis lung involvement is mucolytics to clear respiratory secretions (DNase and inhaled hypertonic saline) and chest physiotherapy [13].

The upward trend of reduction rate upon antibiotics’ effectiveness makes it vital to address new therapeutic alternatives. Products acquired from plants are of interest regarding their health-improving and antimicrobial characteristics. Traditional and complementary medicines are origins of modern and often natural medicines. Out of various traditional classifications of Persian Medicine (PM) is one of the earliest and most precious ones. There is no CF disease in (PM) history, but because of the production of dense excretions in the body ducts, it is set in diseases category owing to the synthesis of thick and gelatinous secretions [14].

The application of Persian Medicine syrup ingredients of “compound honey syrup” (CHS) included Honey, *Crocus sativus* L*, Elettaria cardamomum* (L) Maton*, Zingiber officinale* Roscoe*, Alpinia galanga* (L) Willd*,* and *Cinnamomum verum* J Presl with the common names of being saffron, cardamom, ginger, galangal, and cinnamon, is recommended for thick and sticky secretions of the respiratory tract in the most of (PM) origins [14]. Each of CHS ingredients have anti-inflammatory, anti-microbial and bronchodilator effects and also have CFTR stimulant effects [14, 15].

Honey contains the flavonoid components Quercetin, Kaempferol, Apigenin and Genistein [16, 17]. and phenolic components, which are also among the pharmacological stimulants of CFTR [18]. One hypothesis is that honey, due to its multiplicity of polyphenolic flavonoids, inhibits P-glycoprotein, which is one of the transport proteins in Multi Drug Resistance (MDR) organisms [16]. The components of saffron are crocin, crocetin, picrocrocin, safranal [19] and kaempferol [20]. Saffron has properties such as bronchodilator and anti-inflammatory [21], antiviral [22], anti-asthma effect. The bronchodilator effect of saffron is due to stimulation of beta-2 adrenergic receptors and histamine H1 receptors [22] and inhibition of muscarinic receptors [23].

Cinnamon has anticholinergic, antimicrobial and anti-inflammatory effects [24]. The antibacterial effect of cinnamon on Escherichia coli and Staphylococcus aureus and Pseudomonas aeruginosa has been reported in various studies [25]. Ginger also has anticholinergic, anti-inflammatory and antimicrobial effects [26]. In recent studies, galangal has anti-inflammatory and antimicrobial effects.

Since there have been no previous clinical trials investigating the effect of CHS on FeNO levels in CF patients. Consequently, we initiated this study with the aim of evaluating the effectiveness of CHS on FeNO levels in children with CF.

## Methods

### Patients

In this study, 70 patients were enrolled, and the number of subjects were 44 patients with CF (26 male and 18 female, age: 6–22 years) who were followed-up in Pulmonology Clinic of Mofid Children's Hospital.

### Inclusion and exclusion criteria

All individuals aged 6 and above, who sought care at the Pulmonology Clinic of Mofid Children's Hospital for CF, received a diagnosis from a specialized pediatric pulmonologist. Participation in the research was contingent upon obtaining informed consent from both the parents of children and children themselves, provided they were older than 6 years. Exclusions encompassed CF patients below 6 years old, those necessitating hospitalization due to exacerbation (characterized by increased cough and sputum), individuals with underlying conditions like allergic bronchopulmonary aspergillosis or tuberculosis, those who experienced another acute illness during treatment, individuals with allergies to any CHS components, those who voluntarily opted out of the study, and patients unable to cooperate with the FeNO test.

### Study design

This study was an experimental pretest–posttest evaluation that was conducted on 44 children with CF.

### Drug preparation

CHS is a well-known drink in (PM) which has been utilized for dense and adhesive secretions of the respiratory tract for a long period [10]. In this paper, compound honey syrup was provided with respect to archived pharmaceutical (PM) manuscripts but with modest alterations. Compound honey syrup is an (PM) product which holds a license from the Iranian Food and Drug Administration (IFDA) affiliated to The Ministry of Health of Iran (license number: S-94- 0425). Plants applied in CHS are reviewed as popular therapeutic plants that were provided by Niak Company and were monitored applying standard procedures at quality control laboratory, Niak Company. As a syrup formulation, CHS is a compound of honey, water and an extract of herbs of *Cinnamomum verum* Presl (bark), *Zingiber officinale* Roscoe (root), *Crocus sativus* L (stigma), *Elettaria cardamomum* (L) Maton (fruit) and *Alpinia galanga* (L) Willd (root). Each 100 cm^3^ of compound honey syrup includes extract of saffron, galangal and ginger (1 g), cardamom and cinnamon (2 g) and honey (40 g) [14].

Physico-chemical quality control tests by Niak company on, the amount of impurity, the amount of essential oil and the storage conditions of each component of the sherbet have been carried out separately and finally, tests of appearance characteristics, pH, density, viscosity, dry weight of the extract, microbial and fungal control are performed by Niak Pharmaceutical Company on the final product.

### Clinical evaluation

**Fig. 1:**
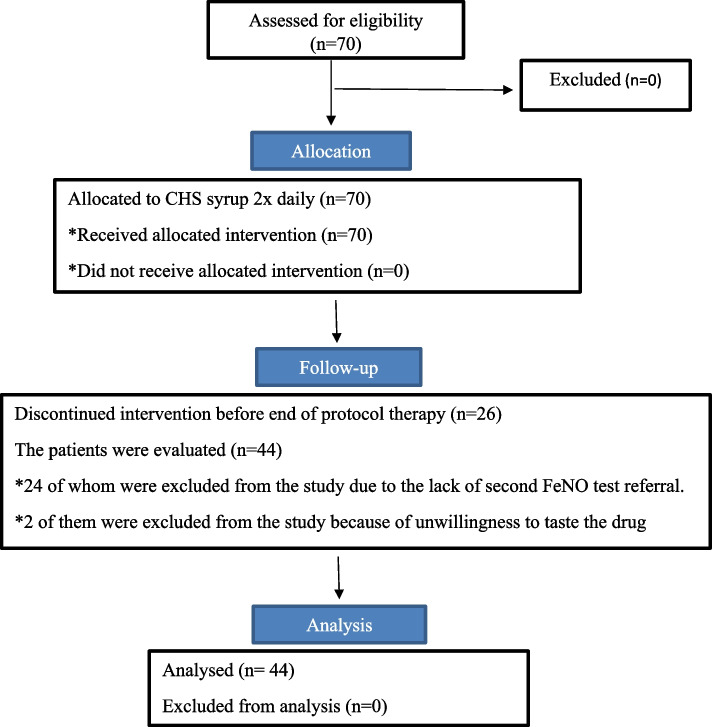
CONSORT flow diagram-modified for non-randomized trial design

Personal data were gathered through taking history and physical examinations of subjects in the time of their visits. During their first visit, the usage of each treatment was described to the subjects who met the inclusion criteria. FeNO test (by the FeNO monitor NO breath®, a product of Bed font UK, which has a diagnostic range below 25 ppb in patients with cystic fibrosis), weight and height were measured. In the next stage, all the standard and required medicines of the patients were prescribed and as an additional treatment, CHS was also prescribed. Patients were instructed on the administration of CHS, with those weighing less than 30 kg or under 12 years advised to take 10 cc per dose, while children weighing over 30 kg or older than 12 years were recommended 5 cc per dose. This medication was to be dissolved in 100 cc of boiled water and taken twice daily, 30 min after a meal. The standard treatment of cystic fibrosis includes the following: chest physiotherapy after nebulizing hypertonic saline, pancreatin enzymes (creon) and fat-soluble vitamins including vitamins A, E, D, K. Follow-up assessments occurred at weeks 2, 4, 8, and 10 via telephone, during which patients were queried about medication usage and potential side effects. Additionally, at weeks 6 and 12, patients received in-person examinations by a pediatric pulmonologist. At the end of week 12, FeNO test was performed (to determine the effect of CHS on the amount of NO in the airways of CF patients), and medication side effects were also asked from patients or their parents to record any side effects Fig. 1.

### Statistical analysis

Data were analyzed by utilizing Statistical Package for Social Sciences (SPSS) version 23 and P-value less than 0.05 was considered statistically significant. The distribution of variables was investigated by using Kolmogorov–Smirnov test. Paired Samples Test was used before and after the intervention to compare the mean and standard deviation of quantitative variables with normal distribution.

## Results

At the beginning of the study, 70 patients were enrolled, 24 of whom were excluded from the study due to the lack of second FeNO test referral, and 2 of them were excluded from the study because of unwillingness to taste the drug. Finally, 44 patients were evaluated. The age of patients were from a minimum of 6 to a maximum of 22 years with a mean and standard deviation of (9.75 ± 3.77). The mean and standard deviation of the highest number of patients were in 6 years old, which was (10.23 ± 3.63) in females and (8.88 ± 3.14) in males, but there was no significant relationship between the two sex groups (*P*. value $<$ 0.159) Table 1.

**Table 1:** Demographic characteristics of CF patients (*n* = 44)

**Variable**		**Mean ± (SD) / Frequency (%)**		***P*-value**
**Age (Year)**	Female	10.23 ± 3.36 / 18 (59.1%)		$<$ 0.159
	Male	8.88 ± 3.14 / 26 (40.9%)		
**Variable**		**Pretest** **Mean ± (SD)**	**Posttest** **Mean** ± **(SD)**	***P*-value**
**Weight (kg)**		29.17 ± 14.07	30.26 ± 14.03	< 0.001
**Height (cm)**		130.31 ± 19.50	131.36 ± 19.42	< 0.001

### The effect of compound honey syrup on patients’ FeNO with cystic fibrosis

The efficacy results are presented in Table 2.

**Table 2:** Changes in FeNO in CF patients before and after CHS therapy

FeNO	Before Mean (SD)	After Mean (SD)	*P*-value
FeNO	10.91 (6.14)	18.14 (11.66)	0.007

At the end of the 12th weeks of the study, FeNO was significantly increased in CF patients (*P*. value < 0.007).

In this study, no dangerous side effects of CHS were detected.

## Discussion

This study demonstrated the prescription of CHS to cause significantly increased FeNO in patients with CF. There was a noticeable difference between the results of FeNO before and after the intervention.

Although in recent years, the use of complementary and alternative medicine has been considered among health care providers and some of the patients with CF use one of the complementary and alternative medicine methods due to its effectiveness upon symptoms reduction in CF patients [27]. In the study by Berger AL et al., which was titled Curcumin Stimulates Cystic Fibrosis Transmembrane Conductance Regulator Cl Channel Activity, it was shown that curcumin increased the activity of both wild-type and DeltaF508 channels. Adding curcumin also increased Cl(-) transport in differentiated non-CF airway epithelia but not in CF epithelia. These results suggest that curcumin may directly stimulate CFTR Cl(-) channels. Also CHS has been used for the improvement of pulmonary manifestation of thick and viscous airway secretions in (PM) [14]. No similar studies were found by searching in reliable scientific databases including PubMed, Scopus, Web of Science and Google Scholar with the keywords Compound honey syrup, Cystic fibrosis, traditional medicine, FeNO and Persian medicine. Anti-inflammatory, bronchodilator, antibacterial effects and CFTR stimulants flavonoid in each components of this product have been performed in the laboratory, animal and in some cases human studies.

The ingredients of CHS also contain flavonoid components which have been shown to stimulate CFTR in various studies, and that include Quercetin, Kaempferol, Apigenin and Genistein in honey [16], Kaempferol in saffron [28], Kaempferol and Quercetin in cinnamon, ginger, cardamom and galangal [16, 29–31]. One study illustrated that 5, 7 and 4 Trimethoxyflavone (TMF) (Apigenin metabolite) is a CFTR activator in both in vivo and in vitro environments [32]. Schmidt et al. in one study demonstrated that pretreatment with Genistein increased the localization of the CFTR protein on the cell surface [33]. R. Melani et al. depicted that Genistein modulates pH, maximal flow and half-activation concentration for CFTR [34], and Chatsri Deachapunya showed that Genistein stimulates chlorine secretion in endometrial epithelial cells by directly activating CFTR through modulating the Tyrosine Kinase-dependent pathway [35]. These studies were performed experimentally and measured the direct effects of flavonoid components on CFTR and the amount of chloride secretion from it, which yielded positive results in the process of improving CFTR performance. As a result of improving the function of CFTR membrane protein, dilution and excretory secretions are seen. Also it displayed decreased NO in the exhalation of patients with cystic fibrosis due to the thick and sticky mucus, high volume of airway secretions, sinus involvement and obstruction of their outlet ducts [16] and underlying CFTR dysfunction [27]. CHS seems to have been able to increase NO excretion in the exhaled air of CF patients due to its stimulant CFTR components. Considering the presence of numerous flavonoids in all components of CHS and the effect of these phenolic components on CFTR, the theory is proposed that CHS can be effective on the performance of CFTR, which, of course, requires more studies to prove this hypothesis.

As mentioned, one of the causes of FeNO reduction in patients with cystic fibrosis is the underlying CFTR dysfunction [36]. Nowadays, studies are designed to produce expensive drugs aiming to influence the CFTR protein. One of these drugs is Ivacaftor. Hartmut G et al. showed that treatment with Ivacaftor increased NO formation in the airways of CF patients (*P*-value = 0.002) [37], while Dornase alpha or hypertonic saline had no effect on NO formation.In this study [37, 38], Ivacaftor was used as a drug that targets CFTR, which could lead to increase NO formation in the airways of these patients. The results of this study were consistent with our study. In 2015, Hartmut G et al. investigated the effect of Ivaceftor on FeNO in 15 patients with CF for 4 weeks. In this study, 8 were adults and 7 were children. The results of this study showed that the amount of FeNO increased significantly, especially in the children group [37]. In 2010, Amin-R et al. level of 15 children with CF, which was not associated with a significant increase in FeNO [39]. Also in 2011, Amin-R et al. investigated the effect of Dornase alfa on 16 children with CF, which was not associated with a significant increase in FeNO [40]. In our study, the effect of CHS on the amount of FeNO was evaluated in 44 children with CF for 12 weeks, which was associated with a significant increase in FeNO. The comparison of these 4 studies shows that Dornase alfa and hypertonic saline have not been effective on the increase of FeNO despite the effect on airway clearance. Therefore, it seems that CHS and Ivaceftor increase FeNO by a mechanism other than airway clearance, which requires more studies in this field**.**

Today, due to the presence of thick secretions in the lungs of patients with CF and the resulting many lung problems, the use of drugs that facilitate the clearance of pulmonary secretions is very important. The excretion of concentrated and purulent airway secretions in patients with CF is a common problem. Analysis of sputum in these patients sdorhows that its high adhesion is towing to macromolecules that include mucosal glycoproteins, modified DNA, and protein polymers [41]. Mucus in patients with CF is rich in extracellular DNA that Dornase alpha, a recombinant human DNase I, can modulate the rheological properties of the secretion through fragmenting airway secretions. Hartmut G et al. outlined that in 83% of cases with Dornase alpha, FeNo changes were consistent with changes in Pulmonary Function Test (PFT), while no similar relationship was seen in the placebo group [38]. It is worth mentioning that simultaneous with the present study, another study was conducted by our research group on the same participants in different stages, the results of which have been previously published. Therefore, the consistency of initial demographic information such as the number of participants, etc. in the two studies is normal [42].

In our study, CHS caused FeNO changes in CF patients according to its ability to dilute thick and viscous secretions especially in the airways.

In general, the results of the present study showed that CHS can be introduced as a complementary treatment with positive effects in increasing the amount of NO in exhaled air and not having significant side effects in CF patients. However, more studies and experiments are recommended to ensure this conclusion.

## Declaration

## Data Availability

The datasets used and/or analysed during the current study are available from the corresponding author on reasonable request.
